# Whole-Transcriptome Sequencing Analysis of Fowl Adenovirus Serotype 4 Infection in LMH Cells

**DOI:** 10.3390/ijms27157028

**Published:** 2026-08-05

**Authors:** Areayi Haiyilati, Xinrui Wang, Xin Miao, Xianglong Wu, Chuake Azhati, Lixia Wang, Li Yang, Qiang Fu, Huijun Shi

**Affiliations:** 1 College of Veterinary Medicine, Xinjiang Agricultural University, Urumqi 830052, China; aray2023@163.com (A.H.); 13201366907@163.com (X.W.); 17832768893@163.com (X.M.); xjau320242826@163.com (X.W.); 13659953079@163.com (C.A.); wlx18299092157@sina.com (L.W.); yl@xjau.edu.cn (L.Y.); fuqiang_xjau@xjau.edu.cn (Q.F.); 2 Urumqi Field Scientific Observation and Research Station for Animal Diseases, Ministry of Agriculture and Rural Affairs Urumqi, Urumqi 830052, China; 3 Xinjiang Key Laboratory of New Research and Development for Herbivorous, Urumqi 830052, China; 4 Xinjiang Regional Key Laboratory of Clinical Veterinary Medicine Research (XJRKLCVMR), Urumqi 830052, China

**Keywords:** LMH cells, FAdV-4, whole-transcriptome sequencing, virus–host cell interaction

## Abstract

Fowl adenovirus serotype 4 (FAdV-4) is the major pathogen responsible for avian hepatitis–hydropericardium syndrome (HHS), which poses a severe threat to the global poultry industry. This study aimed to systematically explore virus–host interactions and elucidate the transcriptomic alterations and underlying molecular mechanisms in LMH cells following FAdV-4 infection. LMH cells were infected with FAdV-4 at a multiplicity of infection (MOI) of 1 for 24 h, and whole-transcriptome sequencing was subsequently performed. Differential expression analysis was conducted between the FAdV-4-infected group and the uninfected Mock group, followed by functional interaction prediction. Quantitative polymerase chain reaction (qPCR) was used to verify the expression profiles of differentially expressed genes (DEGs). The results identified a total of 8292 differentially expressed messenger RNAs (dif-mRNAs), 80 differentially expressed microRNAs (dif-miRNAs), 3263 differentially expressed long non-coding RNAs (dif-lncRNAs), and 52 differentially expressed circular RNAs (dif-circRNAs) in the infected group compared with the Mock group. The identified DEGs were further validated and subjected to Gene Ontology (GO) functional enrichment analysis and Kyoto Encyclopedia of Genes and Genomes (KEGG) pathway enrichment analysis. Additionally, protein–protein interaction (PPI) analysis and regulatory network analyses of lncRNA-miRNA-mRNA and circRNA-miRNA-mRNA were performed. This study provides novel insights and research perspectives into the potential mechanisms underlying FAdV-4–host interactions and further deepens the current understanding of the pathogenesis of FAdV-4 infection.

## 1. Introduction

Hepatitis–hydropericardium syndrome (HHS) is an acute, highly contagious, and immunosuppressive viral disease that seriously endangers chickens [1]. Fowl adenovirus serotype 4 (FAdV-4) is the main pathogen of HHS. The disease first broke out in Angara Goth, Pakistan, and was reported as a new disease in 1987; therefore, it is also known as “Ankara disease” [2,3].

At present, the disease exhibits widespread prevalence across nations globally, resulting in substantial economic detriment and posing a significant threat to the sustainable and health-oriented development of the poultry industry [4]. FAdV-4 mostly occurs in 3-week-old chicks, with a mortality rate of approximately 30–80% [5,6]. During the progression of the infection, clinically affected chickens manifest a pronounced reduction in feed consumption, a depressed state, and the excretion of loose, yellowish mucoid feces [7,8]. Pathological autopsy reveals swelling of the liver and kidneys with typical necrotic foci, and a large amount of pale yellow to dark yellow fluid in the pericardial cavity [9].

Since 2015, HHS outbreaks have occurred in chickens, ducks, and some geese flocks in 28 provinces in China [10,11], including Fujian, Henan, Shandong, Jiangxi, Jiangsu, Shaanxi, Xinjiang, etc. [12]. Over the past decade, the epidemiological pattern in a host of countries and regions, including the Middle East, Russia, Central and South America, Japan and South Korea, has shifted from sporadic cases to recurrent epidemic trends [13,14]. In addition, co-infection of FAdV-4 with other viruses further aggravates the harm of the disease, causing huge losses to the poultry industry around the world [15].

Non-coding RNAs (ncRNAs), primarily comprising microRNAs (miRNAs), long non-coding RNAs (lncRNAs), and circular RNAs (circRNAs), constitute essential components of the transcriptome and exert pivotal regulatory functions during FAdV-4 infection period [16]. Emerging evidence has demonstrated that gga-miR-30c-5p promotes FAdV-4 replication by targeting Mcl-1 [8], while gga-miR-181a-5p facilitates viral immune escape by suppressing the chSTING-mediated signaling pathway [17]. Additionally, lncRNA54128 upregulates its target gene BMP4 to promote FAdV-4-induced apoptosis, and lncRNA loc107053557 acts as a competitive endogenous RNA (ceRNA) by sponging gga-miR-3530-5p, thereby modulating STAT1 expression and inhibiting viral replication [18,19]. Furthermore, circRNAs have been shown to function as molecular sponges within miRNA regulatory networks, influencing viral replication dynamics [20]. These findings collectively underscore the significant involvement of ncRNAs as key regulatory factors in viral infection processes [21].

However, the regulatory functions of transcriptional products in FAdV-4–host interactions remain unexplored through whole-transcriptome analysis. Previous studies mainly focused on the detection of individual transcripts such as lncRNAs, circRNAs and miRNAs, while few investigations have performed combined analysis of multiple RNA components. Therefore, we applied whole-transcriptome sequencing to systematically profile the global gene expression changes in FAdV-4-infected LMH cells for the first time. Our study revealed that both lncRNA-miRNA-mRNA and circRNA-miRNA-mRNA regulatory networks are integral to the molecular regulation of FAdV-4 infection. These findings yield novel insights and offer valuable perspectives for deciphering the intricate mechanisms underlying virus–host interactions.

## 2. Results

### 2.1. Differential Expression Analysis

To explore the functions of mRNA, miRNAs, lncRNAs, and circRNA in FAdV-4 infection cells, we infected LMH cells with FAdV-4 (MOI = 1) for 24 h, and obtained the differential expressions of mRNAs, miRNAs, lncRNAs and circRNAs through high-throughput sequencing combined with Venn diagram analysis: among mRNAs, 912 were encoded by the FAdV-4 genome, 10,469 were co-expressed by FAdV-4 and LMH cells, and 1475 were encoded only by LMH cells; among miRNAs, 36 were encoded by the FAdV-4 genome, 423 were co-expressed by both, and 43 were encoded only by LMH cells; among lncRNAs, 1078 were encoded by the FAdV-4 genome, 3852 were co-expressed by FAdV-4 and LMH cells, and 4863 were encoded only by LMH cells; among circRNAs, 2352 were encoded by the FAdV-4 genome, 1968 were co-expressed by both, and 540 were encoded only by LMH cells.

**Figure 1:**
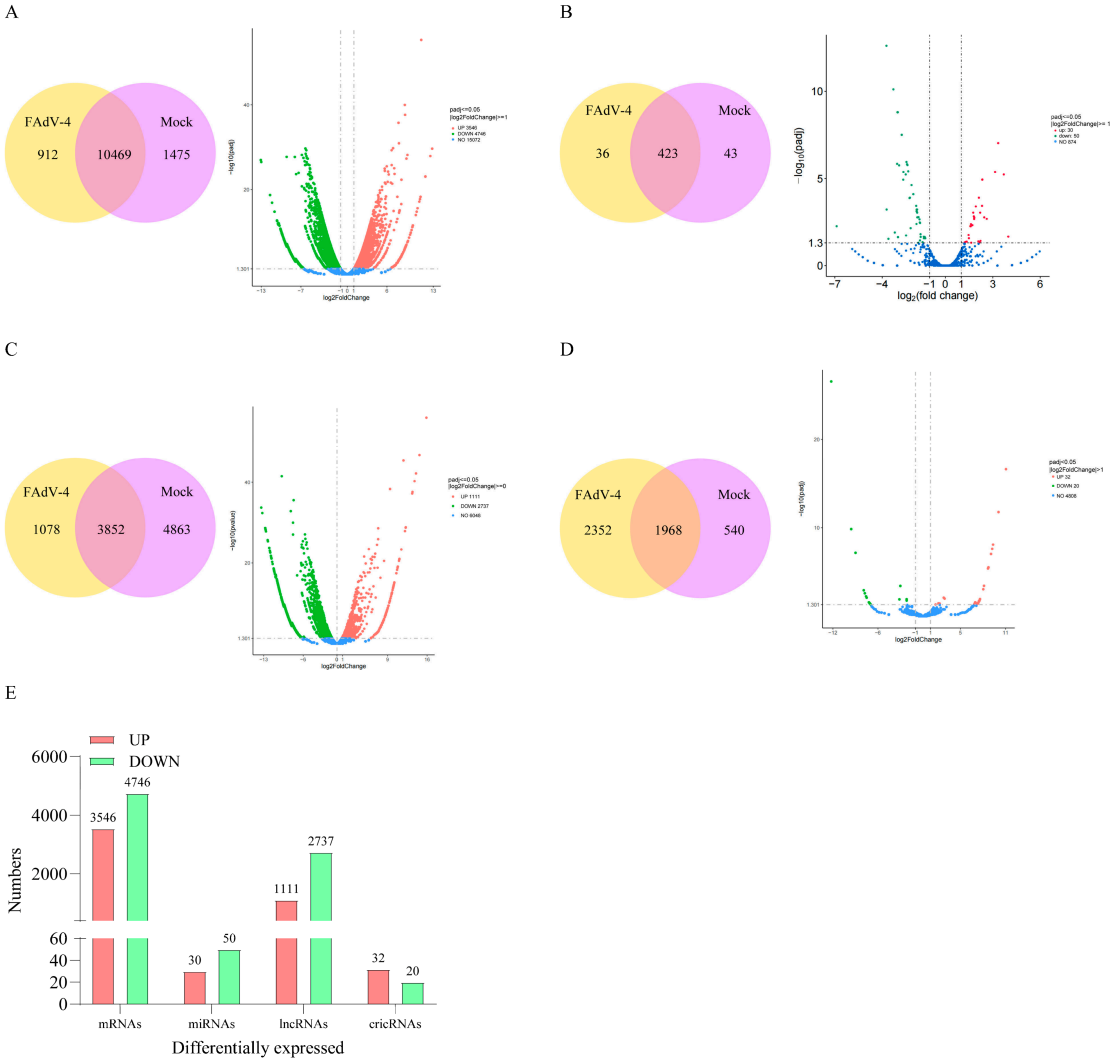
Analysis of gene expression profiles and differentially expressed molecules. (**A**) Venn diagram showing the numbers of mRNAs co-expressed in FAdV-4, LMH cells, and both, along with a volcano plot of differentially expressed mRNAs. (**B**) Venn diagram showing the numbers of miRNAs co-expressed in FAdV-4, LMH cells, and both, along with a volcano plot of differentially expressed miRNAs. (**C**) Venn diagram showing the number of lncRNAs co-expressed in FAdV-4, LMH cells, and both, along with a volcano plot of differentially expressed lncRNAs. (**D**) Venn diagram showing the number of circRNAs co-expressed in FAdV-4, LMH cells, and both, along with a volcano plot of differentially expressed circRNAs (Red indicates upregulation, and green indicates downregulation). (**E**) Statistical chart of the number of differentially expressed molecules, showing the number of upregulated and downregulated mRNAs, miRNAs, lncRNAs, and circRNAs.

According to the screening criteria of padj ≤ 0.05 and |log2FoldChange| ≥ 1, a total of 8292 differentially expressed messenger RNAs (dif-mRNAs) were screened, of which 3546 were upregulated and 4746 were downregulated; a total of 80 differentially expressed microRNAs (dif-miRNAs) were screened, including 30 upregulated and 50 downregulated; a total of 3848 differentially expressed long non-coding RNAs (dif-lncRNAs) were screened, with 1111 upregulated and 2737 downregulated; and a total of 52 differentially expressed circular RNAs (dif-circRNAs) were screened, including 32 upregulated and 20 downregulated (Figure 1).

### 2.2. Validation of Differentially Expressed Genes by qRT-PCR

**Figure 2:**
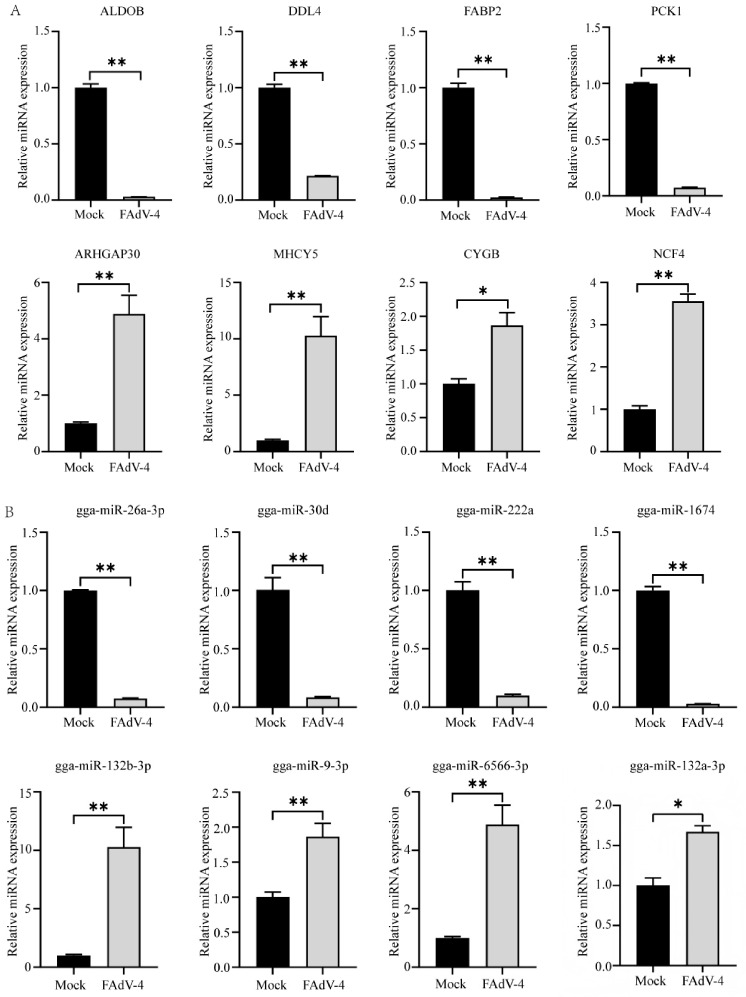

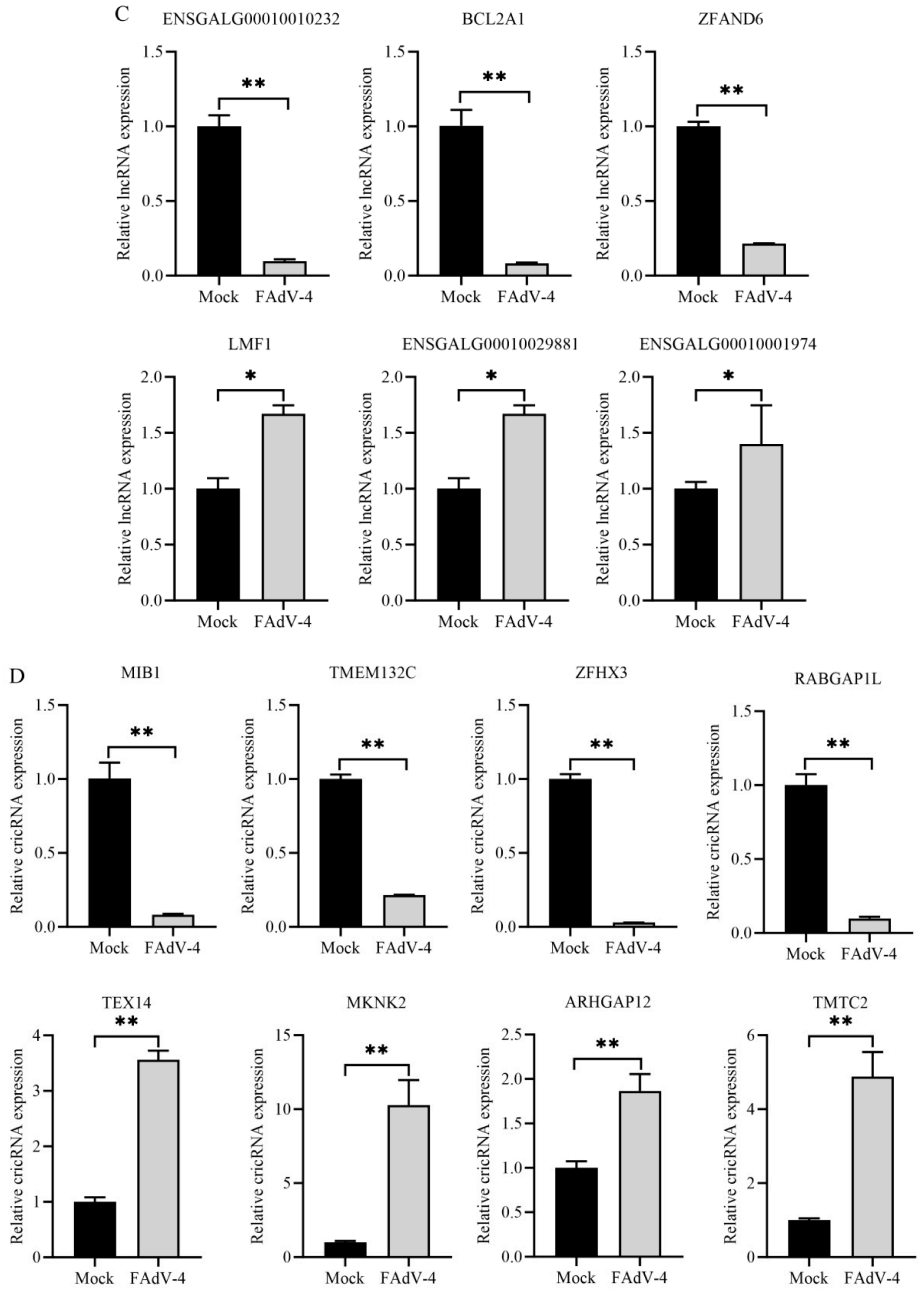
Validation of differentially expressed genes by quantitative real-time PCR. (**A**) Results of differentially expressed mRNAs; (**B**) results of differentially expressed miRNAs; (**C**) results of differentially expressed lncRNAs; (**D**) results of differentially expressed circRNAs. Data are presented as mean ± SD. Compared with the control group, * indicates a significant difference (*p* < 0.05), ** indicates a highly significant difference (*p* < 0.01), and no mark indicates no significant difference (*p* > 0.05).

To validate the accuracy of the sequencing results, we randomly selected differentially expressed mRNAs, miRNAs, lncRNAs, and circRNAs for qRT-PCR analysis. As shown in Figure 2, the alterations in relative transcription levels of the selected RNAs detected by qRT-PCR were closely concordant with the results from whole-transcriptome sequencing, indicating that the sequencing data are reliable and accurate.

### 2.3. GO and KEGG Enrichment Analyses of Differentially Expressed Genes

To better understand the differentially expressed functions and biological processes in FAdV-4-infected cells, we performed GO enrichment analysis to clarify their biological functions. From the results of GO enrichment analysis, the most significant 30 terms were selected to draw a histogram for display. Differentially expressed mRNAs, miRNAs, lncRNAs and circRNAs were mainly enriched in three categories of functions: metabolic regulation, cell movement and structure, and transcription and epigenetics. Non-coding RNAs tend to be involved in transcriptional regulation and maintenance of genome stability and may function by binding to proteins or chromatin modification.

**Figure 3:**
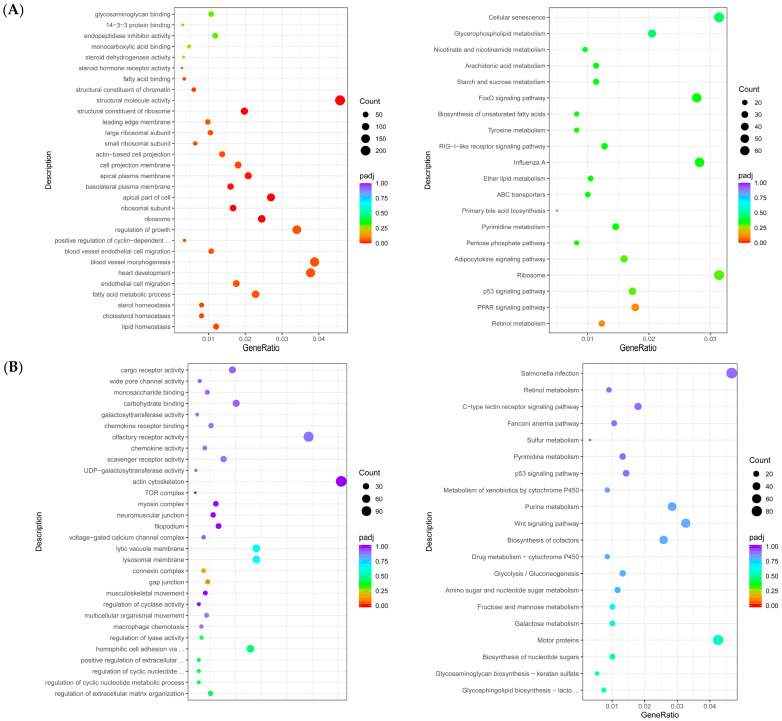

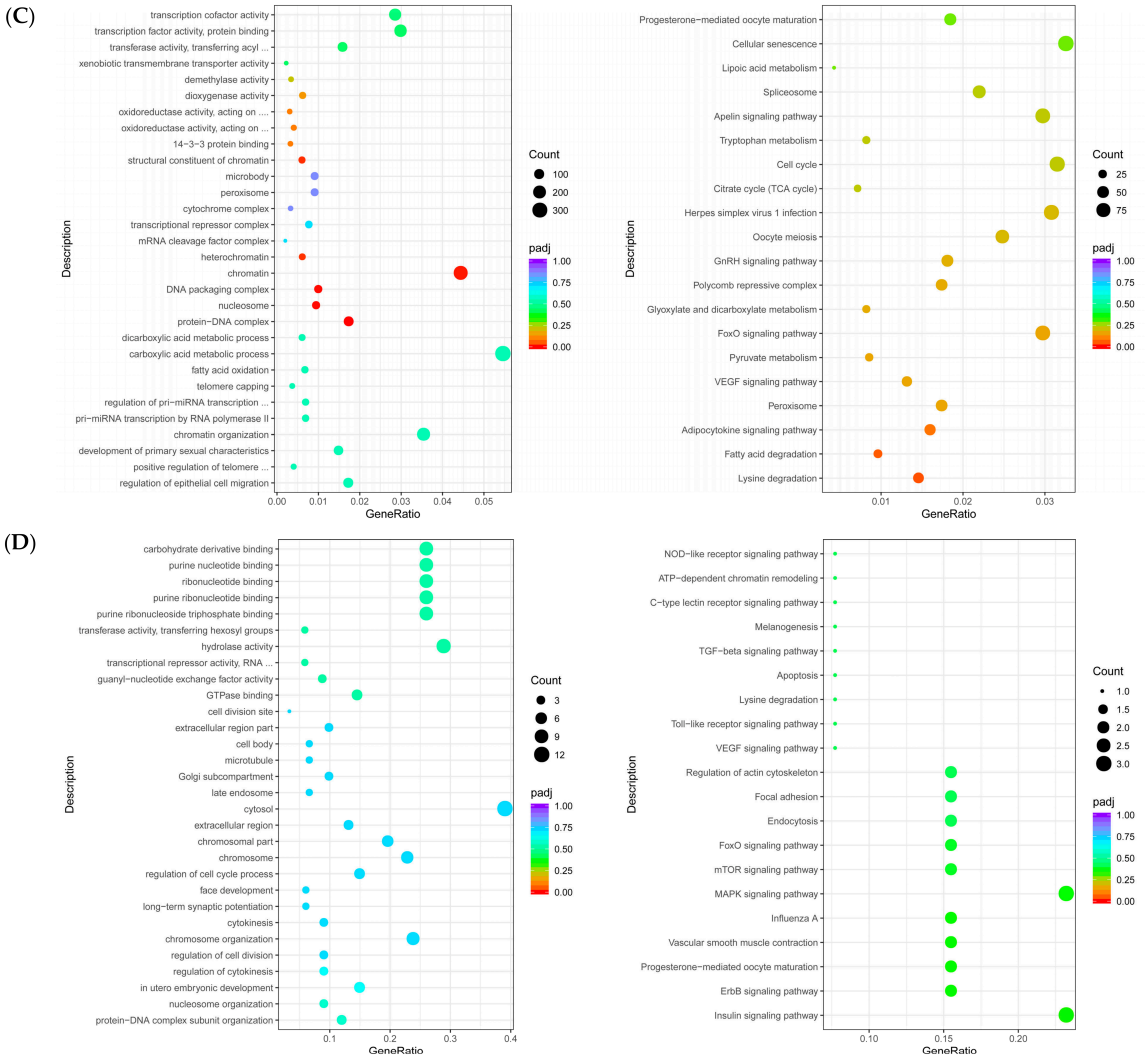
GO-BP functional enrichment and KEGG pathway enrichment analyses of differentially expressed RNAs. (**A**) Scatter plots of GO-BP functional enrichment and KEGG pathway enrichment analyses for differentially expressed mRNAs; (**B**) scatter plots of GO-BP functional enrichment and KEGG pathway enrichment analyses for differentially expressed miRNAs; (**C**) scatter plots of GO-BP functional enrichment and KEGG pathway enrichment analyses for differentially expressed lncRNAs; (**D**) scatter plots of GO-BP functional enrichment and KEGG pathway enrichment analyses for differentially expressed circRNAs. Among them, the GO-BP analysis displays the top 30 significantly enriched GO terms (*p*-value < 0.05) in the biological process category, with the *x*-axis representing the number of enriched genes and the *y*-axis indicating the corresponding functional groups. The KEGG analysis presents the top 20 significantly enriched pathways (*p*-value < 0.05), where the *x*-axis “GeneRatio” denotes the proportion of target genes of differentially expressed RNAs enriched in the corresponding KEGG pathways, and the *y*-axis represents the names of KEGG pathways.

To further analyze the potential roles of these differentially expressed genes (DEGs) in regulatory networks, we performed KEGG pathway enrichment analysis. A total of 163 KEGG pathways were identified for DE mRNAs, 162 for DE miRNAs, 158 for DE lncRNAs, and 34 for DE circRNAs. The enriched KEGG pathways included NOD-like/Toll-like receptor signaling, RIG-I-like receptor signaling, PPAR signaling, TGF-β signaling, mTOR signaling, MAPK signaling, Wnt signaling, p53 signaling, apoptosis, autophagy and ferroptosis signaling pathways. As shown in Figure 3, the top 20 significantly enriched pathways (*p*-value < 0.05) for DE mRNAs, DE miRNAs, DE lncRNAs, and DE circRNAs are displayed. The *x*-axis (GeneRatio) represents the proportion of DEGs enriched in each KEGG pathway, while the *y*-axis (Description) indicates the pathway names (Figure 3). These results imply that the differentially expressed genes may exert critical roles in virus–host interactions.

### 2.4. Protein–Protein Interaction Analysis of Differentially Expressed mRNAs

To elucidate the key host factors modulated by FAdV-4 during infection and to systematically investigate their functional interactions and regulatory relationships through constructing an interaction network, we performed PPI network analysis on all DEGs.

**Figure 4:**
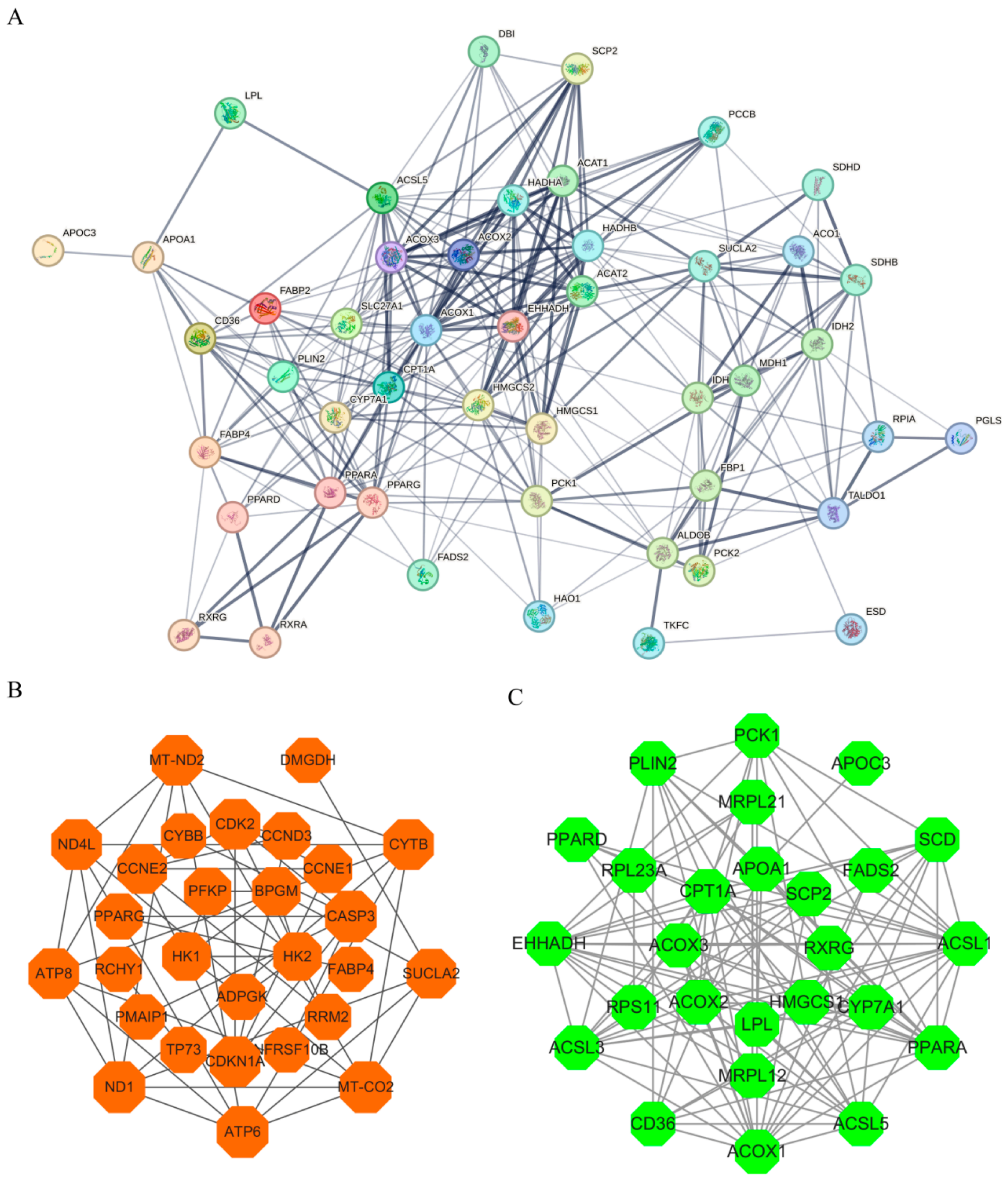
Protein–protein interaction (PPI) network analysis. (**A**) The protein–protein interaction network of differentially expressed genes (DEGs) during FAdV-4 infection was constructed using the STRING database (https://string-db.org/ (accessed on 7 July 2026)). (**B**) PPI network of upregulated differentially expressed genes. (**C**) PPI network of downregulated differentially expressed genes.

The findings indicated that host factors, including members of the PPAR family, ACOX1/2/3, IDH2, and CD36, were positioned at the core of the network, implying that they might serve pivotal functions in the host cell’s reaction to FAdV-4 infection (Figure 4A). Among these, the PPAR family was closely associated with the IFN and NF-κB pathways, while CD36 was strongly linked to the apoptosis process. Additionally, we conducted PPI network analyses on the proteins encoded by the up- and downregulated DEGs. The results showed that the upregulated differentially expressed proteins formed a tightly interconnected network, with HK1/HK2, PPARG, and CDK2 positioned at the core (Figure 4B). Similarly, the downregulated differentially expressed proteins also formed a well-connected interaction network, with the ACOX family, PPAR family, and FASN serving as key components (Figure 4C).

### 2.5. ceRNA Network Construction

**Figure 5:**
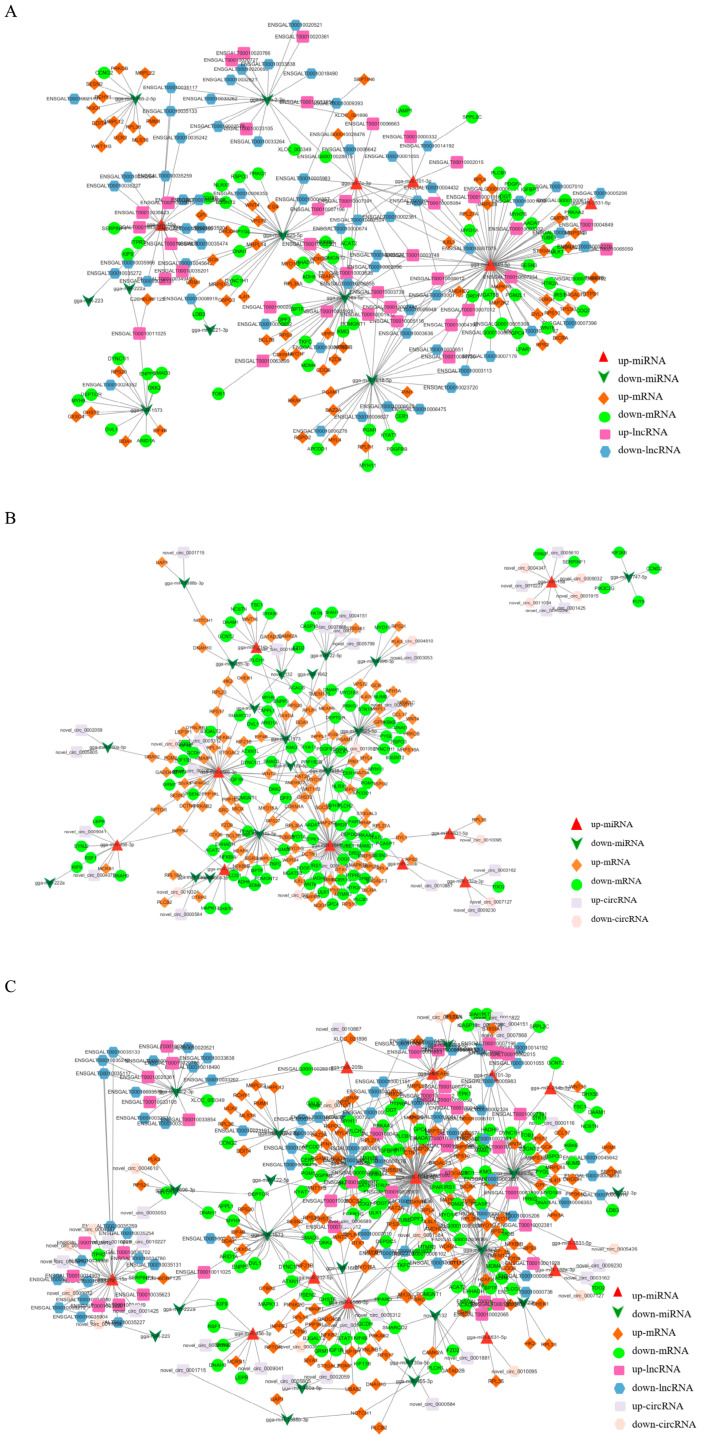
Regulatory networks of mRNA, miRNA, lncRNA, and circRNA. (**A**) lncRNA-miRNA-mRNA network. Red triangles (pointing upward) represent upregulated miRNAs, dark green triangles (pointing downward) represent downregulated miRNAs, orange diamonds represent upregulated mRNAs, green circles represent downregulated mRNAs, purple quadrilaterals represent upregulated lncRNAs, and blue hexagons represent downregulated lncRNAs. (**B**) circRNA-miRNA-mRNA network. Red triangles (pointing upward) represent upregulated miRNAs, dark green triangles (pointing downward) represent downregulated miRNAs, orange diamonds represent upregulated mRNAs, green circles represent downregulated mRNAs, light purple quadrilaterals represent upregulated circRNAs, and pink circles represent downregulated circRNAs. (**C**) Competing endogenous RNA (ceRNA) network. Red triangles (pointing upward) represent upregulated miRNAs, dark green triangles (pointing downward) represent downregulated miRNAs, orange diamonds represent upregulated mRNAs, green circles represent downregulated mRNAs, purple quadrilaterals represent upregulated lncRNAs, blue hexagons represent downregulated lncRNAs, light purple quadrilaterals represent upregulated circRNAs, and pink circles represent downregulated circRNAs.

Based on the co-expression relationships between lncRNA and mRNA, as well as the regulatory interactions between dif-miRNA and dif-mRNA and between dif-miRNA and dif-lncRNA, this study identified significantly differentially expressed lncRNAs and mRNAs that are coregulated by the same miRNAs. For instance, gga-miR-1649-5p was predicted to target MYO1A, PGM2L1, TUBG1, and the lncRNA: ENSGALT0010007176; gga-miR-34a-5p was predicted to target ADH6, RPL35A, COQ6, and the lncRNA: ENSGALT0010063899; and gga-miR-1573 was predicted to target DVL1, EIF4B, DKK1, and the lncRNA: ENSGALT00010011025. Ultimately, an lncRNA-miRNA-mRNA interaction network was constructed to visualize these regulatory pairs (Figure 5A).

Similarly, based on the co-expression relationships between circRNA and mRNA, along with the regulatory interactions of dif-miRNA-dif-mRNA and dif-miRNA-dif-circRNA, differentially expressed circRNAs and mRNAs that are coregulated by shared miRNAs were screened. For example, gga-miR-5631-5p was found to target novel-circ-0010095 and RPL35; gga-miR-6696-3p was probably target to novel-circ-0004610 and PLK3; and gga-miR-22-5p was predicted to target novel-circ-0005799 and FKTN. Finally, a comprehensive circRNA-miRNA-mRNA interaction network was obtained (Figure 5B).

Furthermore, by integrating the lncRNA-miRNA-mRNA and circRNA-miRNA-mRNA networks, we identified differentially expressed circRNAs, lncRNAs, and mRNAs that are coregulated by the same miRNA, and constructed a final ceRNA regulatory network (Figure 5C) to illustrate the crossregulatory relationships among these non-coding RNAs and mRNAs.

## 3. Discussion

HHS is primarily caused by FAdV-4, which is highly prevalent in poultry and leads to substantial economic losses in the industry. Currently, the prevention and control of HHS in China rely mainly on inactivated vaccines; however, this strategy remains insufficient to fully curb the transmission of FAdV-4 infection [22,23]. Therefore, further fundamental research into the pathogenic mechanisms of FAdV-4 is essential for the effective prevention and control of HHS [24]. Nevertheless, the molecular mechanisms underlying FAdV-4 pathogenesis remain poorly understood [25]. The viral life cycle is an obligate intracellular process that entirely relies on the host cell’s machinery, resources, and energy. This intricate dependency encompasses every stage, from initial attachment and entry, through genome replication and transcription, to the final assembly and egress of progeny virions [26]. Transcription is a crucial step in viral biosynthesis. And transcriptome analysis has been widely employed as a research tool to elucidate virus–host interactions. Studies adopting this approach have uncovered that perturbations in cellular gene transcription constitute a pivotal feature during the interplay between FAdV-4 and host cells [27]. Although sporadic studies have reported the roles of certain miRNAs or lncRNAs in FAdV-4 infection, the expression profiles and interaction networks of ncRNAs during infection have not yet been comprehensively characterized through systematic transcriptome sequencing [28].

NcRNAs, including lncRNAs, circRNAs, and miRNAs, have emerged as vital regulators at the host–virus interface, governing processes such as immune evasion, viral replication, and host cell survival [29]. Their functions, mediated through intricate mechanisms including the ceRNAs network and epigenetic modifications, are now considered central to the outcome of viral infections [30]. Among them, lncRNAs and circRNA profoundly influence the dynamic balance between viruses and their hosts by extensively participating in viral gene transcription, host gene transcription, maintenance of mRNA stability, and regulation of mRNA translation, as well as the activation or suppression of host antiviral responses [31]. Meanwhile, miRNAs, a class of shorter ncRNAs, exert negative regulatory effects on gene expression by specifically binding to target mRNAs, thereby promoting mRNA degradation or inhibiting their translation into proteins [32]. Notably, extensive mutual regulatory relationships exist among these ncRNAs [33]. For instance, both lncRNAs and circRNAs can function as competing ceRNAs by sequestering miRNAs, thereby attenuating the inhibitory effects of miRNAs on their target genes and forming intricate post-transcriptional regulatory networks [34]. 

Despite the recognized regulatory significance of ncRNAs, a systematic analysis of the integrated networks encompassing host mRNAs, lncRNAs, circRNAs, and miRNAs during FAdV-4 infection remains absent. Our study is the first to delineate this comprehensive transcriptomic landscape. It not only identified 8292 dif-mRNAs, 80 dif-miRNAs, 3263 dif-lncRNAs, and 52 dif-circRNAs but also GO functional enrichment analysis, KEGG pathway enrichment analysis, and PPI analysis, as well as constructed the lncRNA-miRNA-mRNA and circRNA-miRNA-mRNA regulatory networks involved in FAdV-4 infection. Transcriptomic analyses based on the chicken genomic database have revealed a considerable number of lncRNAs and circRNAs with poorly characterized functions, which may represent novel functional genes or regulatory factors [35]. In studies of avian virus–host interactions, high-throughput sequencing has progressively uncovered the significant role of ncRNAs in viral immune evasion [36]. For instance, avian influenza virus (AIV) infection can activate a circRNA–miRNA regulatory network that modulates host immune responses; similarly, extensive circRNA-involved regulatory pathways have been identified during Newcastle disease virus (NDV) infection, suggesting that viral hijacking of host non-coding RNA networks may represent a common strategy for immune regulation [37]. This mechanism has been further validated in studies on infectious bursal disease virus (IBDV). A genome-wide microarray-based TF–miRNA–mRNA network centered on the transcription factor CTCF was constructed, revealing that the virus suppresses dendritic cell function through epigenetic mechanisms [38]. Furthermore, it demonstrated that the key lncRNA loc107051710, upon viral infection, undergoes nucleocytoplasmic translocation and activates the IRF8-type I interferon-JAK/STAT axis, thereby enhancing interferon-stimulated gene expression and inhibiting viral replication [39]. Research on human viruses, such as adenovirus and influenza virus, has also shown that these pathogens modulate lncRNAs or miRNAs to optimize replication or enhance pathogenicity. Collectively, these findings highlight the universality of viral exploitation of host ncRNAs in immune regulation [40]. The identification of uncharacterized ncRNAs associated with FAdV-4 in this study not only helps to elucidate their potential functions but also furnishes fresh perspectives on avian virus–host interactions, offering a theoretical basis for developing broad-spectrum antiviral strategies targeting ncRNAs. Future research comparing the ncRNA regulatory networks of different avian viruses may further uncover general principles underlying virus–host interactions.

Based on PPI network analysis, this study reveals that FAdV-4 employs a dual strategy during infection to hijack key host cellular pathways, synergistically promoting efficient viral replication. In terms of cell cycle and energy metabolism, FAdV-4 activates the CDK signaling pathway, driving host cells into the S/G2 phase to provide essential DNA polymerase and nucleotide substrates for viral genome replication [41]. Concurrently, the virus upregulates key mitochondrial respiratory chain genes (e.g., MT-ND2), enhancing ATP production to meet the high energy demands of viral factory assembly [42]. This strategy is conserved among multiple viruses; for instance, hepatitis C virus activates the CKI-CDK-Rb-E2F pathway to promote cell cycle progression, porcine epidemic diarrhea virus modulates factors including P21, CDC2, and CDK2/4 to influence the cell cycle, and the hepatitis B virus X protein regulates the host epigenetic state via the p16INK4a-cyclin D1-CDK4/6-pRb-E2F1 pathway, indicating that hijacking the cell cycle is a common mechanism for efficient viral replication [43]. In terms of lipid metabolism regulation, transcriptomic analysis revealed that FAdV-4 infection significantly downregulated multiple key lipid metabolic genes, including the rate-limiting enzyme for fatty acid β-oxidation (CPT1A), peroxisomal oxidases (ACOX1–3), and fatty acid-activating enzymes (ACSL1/3/5). These alterations in gene expression may repress fatty acid degradation and lead to intracellular lipid accumulation, which potentially provides a lipid raft microenvironment for the capsid anchorage of non-enveloped viruses [44]. In addition, the decreased expression of apolipoproteins (APOA1/APOC3) and lipid receptors (CD36/LPL) may reduce the possibility of viral particle neutralization and macrophage recognition [45]. Furthermore, enrichment analysis suggested that FAdV-4 infection may suppress the activity of the PPARA/RXRG nuclear receptor complex and attenuate its positive regulation on fatty acid oxidation. It is speculated that FAdV-4 may reshape host lipid metabolism through this regulatory pattern, thereby potentially facilitating viral structural assembly and immune evasion [46]. In summary, FAdV-4 precisely intervenes in host cell cycle progression and lipid metabolism homeostasis to construct an intracellular environment favorable for viral replication and assembly. This multi-target, coordinated regulatory model affords new perspectives for understanding its pathogenic mechanisms and developing targeted antiviral strategies.

FAdV-4 executes a sophisticated dual strategy of “proliferation promotion and immune defense suppression”, a tactic that not only averts premature host cell lysis but also optimizes the sequestration of cellular resources to facilitate the efficient completion of its replicative cycle. The CDK inhibitor Roscovitine can block viral genome replication, while combination therapy with the PPARA agonist Fenofibrate—through activating lipid catabolic pathways—effectively depletes lipid raft structures essential for viral capsid assembly, thereby inhibiting normal capsid formation [47]. This therapeutic approach restores intracellular lipid oxidative metabolism, removes the structural basis required for capsid assembly, and offers potential targets for interrupting the viral replication cycle. Moreover, APOA1, as a potential neutralizing factor against FAdV-4, warrants further investigation for recombinant protein-based therapy. Through integrated multi-omics analysis, this study successfully delineated the transcriptomic regulatory network during FAdV-4 infection and unveiled a novel mechanism by which the virus hijacks ncRNA core hubs to remodel the host microenvironment. Our sequencing results uncovered that lncRNAs and circRNAs can function as “molecular sponges” to sequester miRNAs, thereby alleviating miRNA-mediated suppression of host apoptosis-related genes and immune regulators. These findings provide an important theoretical foundation for further elucidating the pathogenic mechanisms of FAdV-4.

## 4. Materials and Methods

### 4.1. Cells and Virus

The LMH cell line was provided by Shanghai Meiyan Biotechnology Co., Ltd. (Cat.No: CC-Y4034, Shanghai, China). LMH cells were seeded in 10 cm^2^ dishes and cultured in cell culture medium containing 1× penicillin–streptomycin (Thermo Fisher Scientific, Waltham, MA, USA), 10% fetal bovine serum (Grand Island Biological Company, Grand Island, NY, USA), and DMEM-F12 medium (Grand Island Biological Company). The cells were incubated in an incubator at 37 °C with 5% carbon dioxide concentration.

The FAdV-4 HuBWH strain was initially isolated from the liver of an infected chicken, kindly donated by China Agricultural University (Beijing, China), and stored at −80 °C for later use.

Cells were cultured for 24 h until the confluence reached 80% and then infected with FAdV-4 virus (MOI = 1); uninfected LMH cells served as blank controls. LMH cells infected with FAdV-4 could induce a cytopathic effect (CPE), and the methods for detecting virus titer and viral load refer to the previous literature.

### 4.2. cDNA Library Construction and Whole-Transcriptome Sequencing

Total RNA was extracted from LMH cells and LMH cells infected with FAdV-4 virus (MOI = 1) for 24 h using TRIzol reagent (Thermo Fisher Scientific) according to the manufacturer’s instructions. The resulting RNA samples were then submitted to Novogene Bioinformatics Technology Co., Ltd. (Beijing, China) for subsequent analysis.

MicroRNAs (miRNAs): After passing sample quality control, library construction was performed. Leveraging the unique structures at the 3′ and 5′ ends of RNA (a complete phosphate group at the 5′ end and a hydroxyl group at the 3′ end), total RNA was used as the starting material. Adapters were directly ligated to both ends of the RNA, followed by reverse transcription to synthesize cDNA. The double-stranded cDNA library was generated via PCR amplification. After purification and size selection, libraries with insert fragments between 18 and 40 bp were used for sequencing. Qualified libraries were pooled according to effective concentration and desired sequencing data volume for paired-end sequencing.

Long non-coding RNAs (lncRNAs) and circular RNAs (circRNAs): Ribosomal RNA and linear RNA were removed from the total RNA, and the remaining RNA was fragmented into short segments of 250–300 bp. Using the fragmented RNA as a template and random oligonucleotides as primers, the first strand of cDNA was synthesized. The second strand of cDNA was synthesized using a DNA polymerase I system with dNTPs (dUTP, dATP, dGTP, and dCTP) as substrates. The purified double-stranded cDNA underwent end repair, A-tailing, and adapter ligation. cDNA fragments of approximately 370–420 bp were selected using AMPure XP beads, followed by PCR amplification to complete the library preparation.

Messenger RNA (mRNA): mRNA was enriched from total RNA using Oligo(dT) magnetic beads. After fragmentation, the first strand of cDNA was synthesized using random hexamer primers, followed by second-strand cDNA synthesis. The library was prepared through end repair, A-tailing, adapter ligation, fragment selection, amplification, and purification. The library was quantified using a Qubit instrument (Thermo Fisher Scientific, Waltham, MA, USA) and real-time quantitative PCR, and the fragment size distribution was assessed using a bioanalyzer (Agilent Technologies, Santa Clara, CA, USA).

Library quality control mainly included two methods [48]:

(1) Using AATI analysis to assess library fragment integrity and insert size;
(2) Using qPCR to determine the effective library concentration.

Qualified libraries were pooled according to effective concentration and desired sequencing data volume for paired-end sequencing.

### 4.3. Data Quality Control and Analysis

Raw reads were processed with fastp to remove reads containing adapters, poly-N, and low-quality reads to obtain clean reads. Q20, Q30, and GC content were calculated simultaneously to ensure that downstream analysis was based on high-quality data [49]. HISAT2 was used to construct a reference genome index, and paired-end clean reads were aligned with the reference genome. It can generate a splice junction database using gene model annotation files, and the alignment effect is better than non-splicing tools [50]. String Tie v3.0.3 software was used for new gene prediction, and transcripts were assembled by means of a network flow algorithm and de novo assembly strategy, which are superior to similar software in transcript integrity, accuracy, expression level estimation, and assembly speed [51]. Feature Counts was used to calculate the number of reads mapped to each gene, and FPKM was calculated in combination with gene length (considering the influence of sequencing depth and gene length, it is a commonly used method for estimating gene expression levels at present), systematically completing the quantification of gene expression levels and laying a solid data foundation for the subsequent exploration of molecular mechanisms related to FAdV-4 infection [52,53,54].

### 4.4. Quantitative Real-Time Polymerase Chain Reaction (qRT-PCR)

To validate the transcriptional levels of differentially expressed mRNAs (DE mRNAs) and differentially expressed non-coding RNAs (including DE miRNAs, DE lncRNAs, and DE circRNAs), a relative quantitative approach was employed. Total RNA was extracted from LMH cells using Trizol reagent (Life Technologies Corporation, Carlsbad, CA, USA) and reverse-transcribed into cDNA in accordance with the manufacturer’s instructions. Subsequently, qRT-PCR was performed using SYBR Green Premix (Thermo Fisher Scientific, Waltham, MA, USA) to amplify the target sequences, with primer sets listed in Table 1. The amplification protocol consisted of an initial denaturation at 95 °C for 3 min, followed by 40 cycles of 95 °C for 10 s and 60 °C for 20 s. The relative expression levels of the target genes were calculated using the 2^−ΔΔCT^ method, with glyceraldehyde-3-phosphate dehydrogenase (GAPDH) as the reference gene, and all results were normalized to GAPDH expression.

### 4.5. Differential Expression Gene Analysis

The edgeR (Robinson MD et al., 2010) [55] TMM algorithm was used to normalize the read count data for analysis. Genes with padj ≤ 0.05 and |log2FoldChange| ≥ 1 were identified as differentially expressed genes. Cluster Profiler 3.8.1 software (padj < 0.05) was used for Gene Ontology (GO) and Kyoto Encyclopedia of Genes and Genomes (KEGG) pathway analysis [56,57].

ClusterProfiler software was used to implement GO functional enrichment analysis of differentially expressed genes. Through significant enrichment of differentially expressed genes, padj < 0.05 was used as the threshold for significant enrichment. KEGG is a major public database related to pathway. Pathway significance enrichment analysis takes KEGG pathway as the unit, applies a hypergeometric test to find significantly enriched pathways in candidate target genes, with padj < 0.05 as the threshold for significant enrichment, and analyzes the enrichment of differentially expressed genes in KEGG pathways using cluster Profiler software [55].

Protein–protein interaction (PPI) analysis of differentially expressed genes was based on the STRING database of known and predicted protein–protein interactions. For species present in the database, we constructed networks by extracting target gene lists from the database [58,59].

All raw sequencing reads generated and analyzed in this study have been fully deposited in the NCBI Sequence Read Archive (SRA) public database under the accession number PRJNA1485018 (Bioproject ID: 1485018). The public access link is https://www.ncbi.nlm.nih.gov/bioproject/PRJNA1485018 (accessed on 7 July 2026).

## 5. Conclusions

In this study, we first screened differentially expressed genes in LMH cells following FAdV-4 infection through whole-transcriptome analysis and validated the results. Subsequently, using bioinformatics tools, we conducted protein–protein interaction analysis and constructed lncRNA-miRNA-mRNA and circRNA-miRNA-mRNA regulatory networks. Our findings reveal that miRNA, lncRNA, circRNA, and mRNA exert indispensable roles in virus–host interactions, furnishing fresh perspectives for in-depth investigation of the molecular mechanisms underlying FAdV-4 infection and pathogenesis.

## Figures and Tables

**Table 1:** Primers used in this study.

Primers	Sequences (5′–3′)
ALDOB	CCAAGAGAACGCCAACACCT
	CAGTGCGGAGGAGAGTAGTG
FABP2	GCAATGGGCGTGAATGTGAT
	GCCTGAAAGTTCAGTCCCGT
PCK1	CCAGATAATGGGGAGCCGTG
	CTGCCAGTTAAAGGCCTCGT
DDL4	CACCAAGCCCATCTGTCTGT
	ATGGTCTGTGGTGAGTGCAG
CYGB	GTCAACTTCCCATCGGCCA
	TTCTCTGGGTCGTCGAGGTT
MHCY5	CACTCCCTGCGCTACTTCAA
	GGCCTTCTTGGTCTCTGCAT
ARHGAP30	GTAAAAGAGGAGAGGGGCGG
	GGTGGCACCTCCAAAGGTTG
NCF4	ACAGCTGCGAGAAAAGAGTGA
	ACTTTCCCTGGCAATACGGG
gga-miR-30d	GTTGCTGTAAACATCCCCGAC
	TTTCAGTCAGATGTTTGCTGC
gga-miR-222a	GTAGTTGCCCATCAATCGCTC
	CTCTGATGACATCTCATATCT
gga-miR-1674	GGTAAGAGCATTCCTGCAGG
	GGTGCTGGCAGGCTTGGGCT
gga-miR-26a-3p	GTCACCTGGTTCAAGTAATC
	GGTTACTTGCACTGGGAGGC
gga-miR-132b-3p	ACCATGGCTGTAGACTGTTA
	CAATCTAAAGCCACGGTCG
gga-miR-9-3p	TTGGTTATCTAGCTGTATG
	GTAAAAACCCGCTCGC
gga-miR-6566-3p	GAGCCGTCTTTGTCCGAGAAG
	GCTCCGGACGTTCTGCA
gga-miR-132b-3p	ACCATGGCTGTAGACTGTTA
	CAATCTAAAGCCACGGTCG
ZFAND6	CTTTGTTCCACTGGTTGCGG
	TGCACTGAACCGGTAAGGAC
BCL2A1	CACGGAGTTCAGCTCACTGG
	CCAGCCACCGTTTGCATCTA
ENSGALG00010010232	CAGAGCAGTGTGGAGCAGAG
	TGTGAGCAAGGTTCAGGTCA
ENSGALG00010001974	TGGACAGCAGGTCCATTTCC
	TGGCAGAGTGGCAGTGAAA
ENSGALG00010029881	CGCTGGAACGGCCCTGCGGTC
	GGACTGGGAGCAGGCATCTG
LMF1	ACTTCATCGAGCTGGTGGTG
	CTGCAGCACACGATCCTTCA
TMEM132C	CTGGTGGAAGGACAGACCAC
	CTGAGCACTGCCATTGAGGA
MIB1	GAAGGTGACACTCCGCTTCA
	CGGTCGTGGTAACTTGGACA
RABGAP1L	CGTGGTTCACCAGCAAACTG
	AGGGTCTAAAACAAGTTGCAAGG
ZFHX3	TCACACAGTGAACTCGAGGC
	ATCTGTCCCAGGTCCTCCTC
ARHGAP12	AGCCCTGGTCAAACTCTTGG
	GCTTGGTGGAGTAGTTGGCT
MKNK2	GGAGATGCTCTACCAGTGCC
	CCTTGTTGTGCAGGAAGTGC
TMTC2	CAGTGGGAGCCACAAGTCAT
	GAAGAGGCAGGCTCCAATGT
TEX14	AGGTTTGGTGGCCTTGTTCA
	GATGGGGCTGAAAGCTGAGT

## Data Availability

All raw sequencing reads generated and analyzed in this study have been deposited in the NCBI Sequence Read Archive (SRA) database under accession number PRJNA1485018, ID: 1485018 (https://www.ncbi.nlm.nih.gov/bioproject/PRJNA1485018 (accessed on 7 July 2026)).
